# Obesity and its impact on COVID-19

**DOI:** 10.1007/s00109-021-02072-4

**Published:** 2021-04-06

**Authors:** Angélica J. M. de Leeuw, Maureen A. M. Oude Luttikhuis, Annemarijn C. Wellen, Christine Müller, Cornelis F. Calkhoven

**Affiliations:** 1 University Medical Center Groningen (UMCG), University of Groningen, 9700 AD Groningen, The Netherlands; 2 European Research Institute for the Biology of Ageing (ERIBA), University Medical Center Groningen, University of Groningen, 9700 AD Groningen, The Netherlands

**Keywords:** Obesity, SARS-CoV-2, COVID-19, Immunopathology, RAAS

## Abstract

The severe acute respiratory syndrome-coronavirus-2 (SARS-CoV-2) pandemic has proven a challenge to healthcare systems since its first appearance in late 2019. The global spread and devastating effects of coronavirus disease 2019 (COVID-19) on patients have resulted in countless studies on risk factors and disease progression. Overweight and obesity emerged as one of the major risk factors for developing severe COVID-19. Here we review the biology of coronavirus infections in relation to obesity. In particular, we review literature about the impact of adiposity-related systemic inflammation on the COVID-19 disease severity, involving cytokine, chemokine, leptin, and growth hormone signaling, and we discuss the involvement of hyperactivation of the renin-angiotensin-aldosterone system (RAAS). Due to the sheer number of publications on COVID-19, we cannot be completed, and therefore, we apologize for all the publications that we do not cite.

## The obesity pandemic

The worldwide prevalence of obesity increased 3-fold between 1975 and 2016, with 13% of adults being obese and 39% being overweight in 2016 (source: World Health Organization (WHO), https://www.who.int). If this incline continues, it is estimated that in 2030, 38% of the population worldwide will be overweight and 20% will be obese [1]. Obesity is defined as a too high body weight compared to height. The WHO distinguishes three groups: class I (BMI 30.034.9 kg/m^2^), class II (35.0–39.9 kg/m^2^; severe obese), and class III (BMI >40.0 kg/m^2^; morbid obese) (https://www.who.int).

Excess energy from the diet is stored as fat in the white adipose tissue (WAT), distributed widely throughout the body. WAT is subdivided in two major categories; visceral fat depots (around abdominal viscera in mesentery and omentum) and subcutaneous fat depots (under the skin). In addition to fat-storing adipocytes, WAT contains immune cells. Together, they affect whole-body homeostasis through metabolic, endocrine, and immune functions. Chronic overnutrition and resulting obesity cause severe derangements in these functions, associated with increased leptin secretion, local inflammation, and release of inflammatory mediators that may negatively affect the function of other tissues [2]. This low-grade inflammatory state is a major risk factor for developing diseases such as diabetes mellitus type 2, hypertension, cardiovascular diseases, and fatty liver disease [2–5]. The health consequences of obesity are linked to the side of fat storage with the accumulation of visceral fat being associated with more adverse health outcomes, inflammation, and metabolic syndrome, as opposed to the healthier fat accumulation in the subcutaneous depots [6, 7]. Excessive fat in WAT is stored largely without the number of adipocytes increasing, resulting in adipocyte hypertrophy, which is associated with reduced oxygen supply and hypoxia, and an increase in macrophage infiltration and inflammation [8–10]. Since the storage capacity of the hypertrophic cells is limited, fat starts to accumulate in ectopic tissues such as the liver, heart, and skeletal muscles [8]. This dyslipidemia further promotes metabolic disorders. However, an exception to this scenario exists as individuals who are chronically obese but stay—at least transiently—metabolically healthy. Evidence from mice and a few human studies suggests that storing a surplus of nutrients as fat through adipocyte hyperplasia (an increasing number) is associated with metabolic health [11]. The fat storage can be distributed over more fat cells; the individual fat cells stay smaller, are metabolically more active, and are less inflamed [6]. So far, little is known about the underlying molecular mechanisms driving fat storage in either the hypertrophic or the hyperplastic direction. An exception is the function of the metabolic transcription factor C/EBPβ which was linked to hypertrophic versus hyperplastic fat accumulation in mice with a high-fat diet [12]. Such regulators may be attractive targets to therapeutically prevent metabolic and immunological complications associated with obesity.

## The SARS-CoV-2/COVID-19 pandemic

In December 2019, health authorities in China reported an outbreak of pneumonia of unknown cause in Wuhan, Hubei Province, China. The first reported cases were linked to a local seafood market in Wuhan City, shortly after the causative coronavirus was isolated from lower respiratory tract samples of pneumonia patients. The virus was named severe acute respiratory syndrome coronavirus-2 (SARS-CoV-2), and the disease it causes was called Coronavirus Disease 2019 (COVID-19). Human-to-human transmission is well established in SARS-CoV-2 and the virus is mainly transmitted through respiratory droplets and direct contact. Infected patients may be asymptomatic or show symptoms such as high fever, chills, loss of smell and taste, cough, shortness of breath, or difficulty in breathing. The most reported symptoms at the onset of disease are fever (87.9%), cough (67.7%), and fatigue (38.1%) [13–15]. The respiratory symptoms can be mild and consist of dry cough, shortness of breath, or rhinitis. In the more severe COVID-19 cases, these symptoms can progress to bilateral pneumonia and acute respiratory distress syndrome (ARDS). Furthermore, multi-organ dysfunction including acute cardiac and kidney injuries, arrhythmias, gastrointestinal, and liver function abnormalities, and thromboembolic complications have been documented in infected patients. Management of severe disease progression usually requires admission to the intensive care unit (ICU) and is associated with high mortality of ∼30–40% [16–18]. The viral incubation period ranges from 2–14 days with an average of 5.2 days [13]. The diagnostic method of choice is the detection of viral genetic material in a nasopharyngeal swab or sputum sample using polymerase chain reaction (PCR), more recently supplemented with detection of SARS-CoV-2 antigens with antibody-based tests. An additional diagnostic tool is a computer tomography (CT) scan of the chest which shows ground-glass opacities, interstitial infiltration, or multiple patchy consolidations in both lungs [19]. Due to globalization, the virus was able to spread rapidly worldwide and on March 11, 2020, the WHO officially declared COVID-19 a pandemic. As of March 2021, COVID-19 caused more than 120 million reported infected cases and around 2.7 million reported deaths worldwide (https://www.who.int). Although no effective antiviral drugs exist, vaccines against SARS-CoV-2 are now available and world-wide vaccination programs are expected to be completed during 2021.

## The biology of coronaviruses

**Fig. 1:**
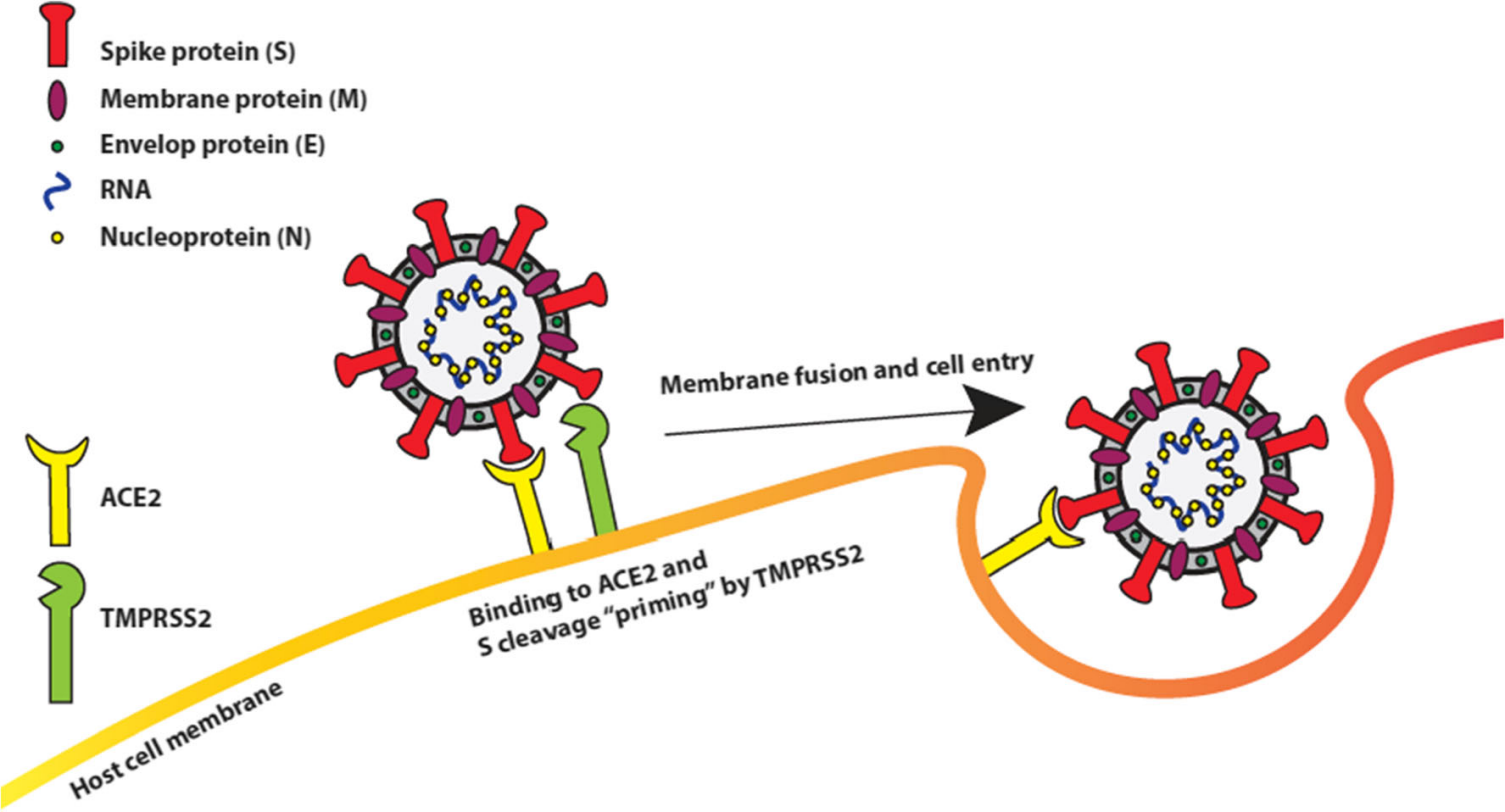
SARS-CoV-2 and cellular components in viral infection. The spike (S), nucleocapsid (N), membrane (M), and the envelope (E) proteins make up the main structural proteins of SARS-CoV-2. The cellular angiotensin-converting enzyme 2 (ACE2) exopeptidase acts as a receptor for SARS-CoV-2 spike protein (S) binding. Subsequent cleavage of S by the cellular transmembrane serine protease 2 (TMPRSS2) results in membrane fusion of the virus and its cell entry

The family of coronaviruses consists of two subfamilies; the Totovirinae and the Coronavirinae [20]. The Totovirinae mainly cause disease in terrestrial and aquatic animals. The Coronavirinae, in particular the genus of betacoronaviruses, cause a wide variety of diseases in mammals, differentiating from the common cold to more severe respiratory diseases like SARS and MERS in humans [21]. Coronaviruses share a couple of important features contributing to transmission, viral replication, and viral immunopathology [22]. Coronaviruses have a non-segmented, single-stranded positive-sense RNA-genome, and with approximately 30.000 bases in length, they are the largest known mature RNA-molecules in biology [23, 24]. The virus genome codes for four main structural proteins; the spike (S), nucleocapsid (N), membrane (M), and the envelope (E) proteins as well as for other non-structural proteins required in viral replication [23]. Of the structural proteins, the S-protein is a critical surface-located trimeric glycoprotein which mediates attachment to host cell receptors and viral entry [22, 25–27]. The N-protein’s function is to package the viral RNA-genome into a ribonucleoprotein complex [28]. The M-protein is the most abundant protein and shapes the viral envelope [29]. The smallest structural protein is the E-protein which has important roles in virus production and maturation [30, 31] (Fig. 1).

Both SARS-CoV and SARS-CoV-2 enter host cells by binding to Angiotensin-converting enzyme 2 (ACE2) through S-proteins on their surface [22, 32], yet SARS-CoV-2 binds ACE2 with a considerably higher affinity than SARS-CoV [33]. Based on inter-species differences in the ACE2 gene and modeling of S-protein ACE2-complex structure predictions, SARS-CoV-2 is predicted to infect a broad range of animals, excluding some fish, birds, and reptiles [34]. The S-protein contains two subunits, S1 and S2. The first step of virus entry is binding of the S1 subunit, via its N-terminal portion, into a hydrophobic pocket in ACE2. Next, the S-protein is cleaved by the host type II transmembrane serine protease TMPRSS2 and possibly also by lysosomal cathepsins like furin [35]*.* The cleavage causes the S1 and S2 subunits to stick to each other, inducing dissociation of the S1 subunit with ACE2 and a change in S2 structure causing a stable post-fusion state. This process, called priming, facilitates the fusion between the viral and cellular membrane and the entry of the virus into the cell [36]. The S-proteins of SARS-CoV and SARS-CoV-2 differ by approximately 380 amino acids (the viral genomes show 79.6% similarity) [37], including five to six vital amino acids which cause 10 to 20-fold higher binding affinity of SARS-CoV-2 for ACE2. The stronger SARS-CoV-2 S-protein-ACE2 interaction is responsible for the high pathogenic nature of COVID-19 [16, 17, 38, 39]. ACE2 is widely expressed throughout the body with a high expression in the lungs, intestines, and adipocytes in WAT [40]. Some studies suggest the ACE2 receptor is upregulated by obesity, cardiovascular disease, and smoking [41, 42], while other studies suggest cellular levels of ACE2 are the same in adipocytes of obese and non-obese individuals [43]. Nonetheless, experiments in rats have shown upregulation of ACE2 in adipose tissue in response to a high-fat diet [44]. Taking into account the much higher volume of adipose tissue, it is reasonable to assume increased quantities of ACE2 in adipose tissues of people with obesity.

During infection with SARS-CoV/-2, the S-protein-ACE2 complex is internalized in the cell resulting in a loss of functional, membrane-bound ACE2. As we will discuss below, in a normal situation, ACE2 has anti-inflammatory effects and is considered to act protective in case of acute respiratory distress syndrome (ARDS). This protective mechanism is compromised through ACE2 receptor downregulation in SARS-CoV/-2, resulting in more prominent ARDS [38].

Since late 2020, mutations of SARS-CoV-2 are starting to emerge. A few variants first spread rapidly among populations in Great Britain (lineage B.1.1.7), South Africa (lineage 501Y.V2), and Brazil (lineages B.1.1.28.P1/P2), of whom they thank their common names [45]. These now world-wide present variants mainly contain mutations in the spike (S) protein that affect the ACE2 binding domain and other parts of the protein. It has been proposed that these mutations result in increased ACE2 binding and internalization of the virus leading to higher transmission rates of all three SARS-CoV-2 variants [46]. Moreover, infection with the British variant is associated with an increased risk of hospitalization and mortality in the UK and Danish populations [47–49]. In addition, mutations found in the South African and the Brazilian variant may affect the recognition and neutralization by antibodies and thereby promote immune evasion, which could result in higher re-infection rates as has been suggested for the Brazilian variant [50–52]. How these mutations affect people with obesity is not yet clear, and awaits completion of studies currently pursued.

## COVID-19 immunopathology

In 10–20% of COVID-19 patients, a second more severe phase of the disease develops typically 7 to 14 days post-infection. This phase is characterized by respiratory failure and acute respiratory distress syndrome (ARDS), as well as multi-organ failure, which ultimately can lead to death [53–55]. Deregulated innate and adaptive immune responses are believed to be responsible for the life-threatening development of the disease. The most prominent feature is a hyper-inflammatory response with strongly increased inflammatory markers in the blood of patients, such as C-reactive protein (CRP), ferritin, inflammatory cytokines, particularly Interleukin (IL)-6, IL-1β, tumor necrosis factor α (TNF-α), and chemokines like CCL2, CCL3, and CCL5 [56–62]. An increased accumulation of macrophages and other phagocytic immune cells was found in the lungs of patients with severe disease [63, 64]. Studies suggest that activated macrophages provoke further attraction of innate immune cells through the release of inflammatory cytokines, which amplifies the secretion of inflammatory cytokines causing failure to terminate the inflammatory response, a condition termed cytokine storm syndrome (CSS) [65, 66]. High concentrations of inflammatory cytokines and chemokines can induce apoptosis of epithelial and endothelial cells of the lungs and leakage of vasculature which ultimately results in severe lung damage observed in patients with ARDS [65]. Effective treatment with anti-inflammatory drugs like dexamethasone or hydrocortisone (https://www.recoverytrial.net) [67, 68] or the anti- IL-6 receptor antibody tocilizumab (https://www.remapcap.org) [69–71] shows that the uncontrolled inflammatory response is an important contributor to severe disease progression. Furthermore, a strong increase in numbers of immature neutrophil precursors and the occurrence of dysfunctional mature neutrophils have been observed in the blood of patients with severe COVID-19 disease probably reflecting a defect in myelopoiesis [72, 73]. If and how these cells contribute to the health deterioration of COVID-19 patients is currently unclear. Both in mild and severe COVID-19 cases, a decrease of lymphocytes in the blood has been observed, particularly of natural killer (NK) cells and T-cells. Within the T-cell compartment, CD8+ cytotoxic T-cells seem to be more strongly affected [74]. In patients with severe disease progression, this lymphopenia is more pronounced. In addition, molecular signs of T-cell exhaustion are detectable particularly in severe cases suggesting that anti-viral T-cell functioning is compromised [75, 76]. It is not completely understood what underlying causes are responsible for lower numbers and exhaustion of T-cells in severe disease cases. It has been proposed that COVID-19 infection can induce apoptosis signaling pathways in lymphocytes [77]. In addition, macrophages induce lymphocyte apoptosis through Fas/Fas ligand interactions or lymphocyte necrosis through high levels of secreted IL-6 [78]. Furthermore, it has been suggested interferon (INF) type I response is impaired in patients with severe disease progression but not in patients with mild symptoms. Type I interferons are secreted from infected cells, pathogen-sensing macrophages, and dendritic cells, and induce interferon-stimulated genes (ISGs) in the target cells mediating antiviral response. Thereby, type I interferons activate both the innate and adaptive immune system; they furthermore enhance antigen presentation and induce a defense mechanism in cells neighboring the infection site to limit viral spread. INF-α levels in circulating white blood cells were found to be strongly decreased particularly in severe COVID-19 cases similar to the INF response gene signature, suggesting that mounting of an anti-viral response is dampened in severe cases [79]. However, other data contradict these findings showing that patients with severe disease progression can have a functional INF type I response [62, 80, 81]. It was even suggested the INF I response could increase the inflammatory state in patients with severe COVID-19 [82]. Thus, it is not clear if and to which extend failure to mount an INF type I response contributes to the severe progression of the disease.

## Obesity and COVID-19

Soon after the COVID-19 outbreak, worldwide observations revealed 70–90% of SARS-CoV-2 infected patients suffering from respiratory failure who were admitted to the ICU are overweight [83–85]. Since then, multiple studies revealed a strong correlation between ICU admission and BMI, independent of other (metabolic) risk factors. A Dutch study showed 90% of the SARS-CoV-2 infected patients with respiratory failure had a BMI higher than 25 kg/m^2^ and a mean BMI of 30 kg/m^2^ and that the severity of the disease increases significantly with BMI [86]. Concurrently, COVID-19 patients requiring mechanical ventilation in a Seattle-based cohort had a mean BMI of 33 kg/m^2^ [87]. A Southern California–based study linked BMI >40 to greater relative risk most strikingly among patients aged 60 years or younger and in men. [88]. An international multicenter retrospective cohort study revealed a linear association between BMI and the need for invasive mechanical ventilation (IMV) for critically ill COVID-19 patients, which was more pronounced in younger females and independent of other metabolic risk factors. In addition, a non-linear significant association between BMI and mortality was observed in obesity class III (≥40 kg/m^2^) [89].

Altogether, the studies show that progression of obesity to morbid obesity increases the risk for mechanical ventilation and admission to the ICU by more than 2-fold and is significantly linked to being male and having a high BMI. Obesity is also known as an independent risk factor for disease severity and mortality in the 2009 H1N1 influenza pandemic [90, 91]. Longer ICU length of stay was observed in infected obese individuals even if they did not suffer from chronic conditions which would have increased the risk of influenza-related complications [92].

Mechanically, obesity impairs pulmonary function characterized by a decline in expiratory reserve volume, functional capacity, and respiratory system compliance. Abdominal obesity jeopardizes these functions even more in the supine position because of decreased diaphragmatic excursion [93]. Nonetheless, the so-called obesity paradox is a phenomenon of reduced mortality with septic shock or sepsis and ARDS in patients who are overweight or obese [94, 95]. A possible explanation for this phenomenon is the obesity-related chronic pro-inflammatory milieu functions as a protective environment limiting a second, more aggressive infection, such as sepsis or ventilation-induced infections [96]. In the case of COVID-19, however, a high BMI is related to a higher risk of developing respiratory failure and thus the need for mechanical ventilation [86, 87]. Normally, obese patients are admitted to the ICU with mechanical hypoventilation as a result of hypercapnic respiratory failure, but in SARS-CoV-2 infection, the clinical presentation is hypoxic respiratory failure. These findings suggest COVID-19 elicits an unusual response. Therefore, even though obesity-associated comorbidities contribute indirectly to a severe disease course, it seems there is an independent association between obesity and COVID-19 severity. Several recent studies suggest that the accumulation of visceral fat and immunological alterations associated with obesity may be involved in developing a more severe disease course.

## Obesity and the innate immune system

**Fig. 2:**
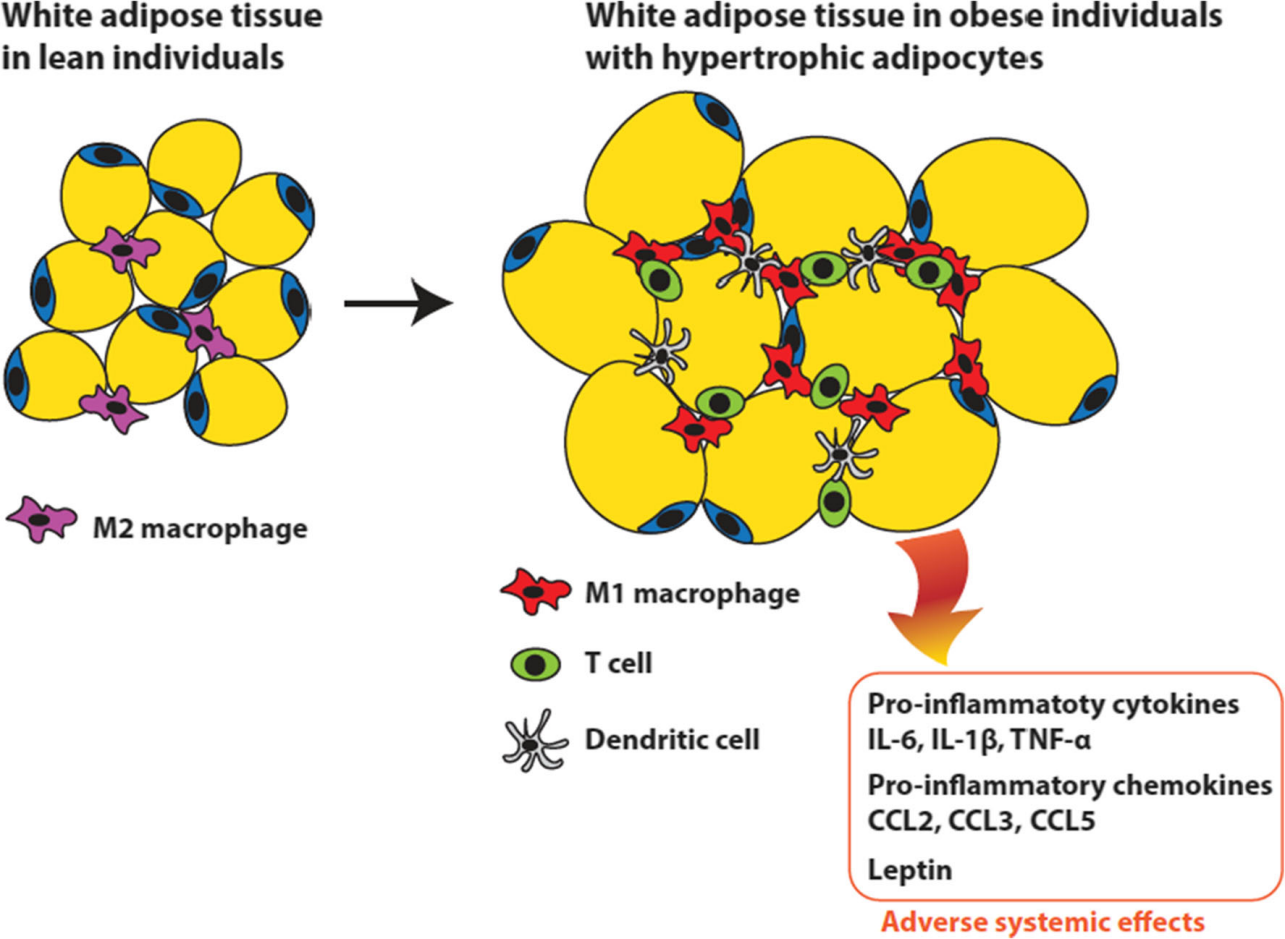
Adverse systemic effects of fat accumulation in hypertrophic adipocytes. In obese individuals, a surplus of energy is stored as fat in hypertrophic adipocytes in the WAT. The hypertrophic fat cells secrete chemokines causing infiltration by immune cells and polarization of resident macrophages to the pro-inflammatory M1 type, resulting in elevated secretion of pro-inflammatory cytokines. In addition, high leptin secretion associated with leptin resistance in obese individuals further contributes to the inflammatory phenotype. Together, these processes elicit adverse systemic pro-inflammatory effects that aggravate COVID-19 symptoms. Figure based on Fig. 2 in [99]

Hypertrophic adipocytes, in particular in the visceral WAT, recruit polarized macrophages that induce low-grade systemic inflammation through the production of excessive amounts of inflammatory cytokines, of which IL-6, TNF-α, IL-1, IL-10, and MCP-1 are the most important (Fig. 2) [90, 97]. Infection with SARS-CoV-2 may provoke a cytokine storm, characterized by a similar set of cytokines (IL-6, TNF-α IL-2, IL-7, INF-Y, MCP-1) [90, 97]. The cytokine IL-6, in particular, appears to be associated with a more severe disease course, as higher levels of IL-6 were observed in non-survivors compared to survivors of COVID-19 [97]. Adipose tissue can act as a reservoir for the production and secretion of IL-6 and hence amplify the cytokine storm and contribute to higher mortality in COVID-19 [98]. In addition, the high cytokine levels secreted from hypertrophic fat cells into the bloodstream, including IL-6, counteract the termination of the anti-viral immune response in the lungs of and thereby promote a cytokine storm in COVID-19 patients with a severe disease development.

## Obesity and the adaptive immune system

In addition to the innate immune system, obesity also affects the adaptive immune system. The activation and function of CD4+ and CD8+ T-cells are diminished in people with overweight or obesity. Metabolic changes in T-cells associated with an impaired memory T-cell response were observed, which makes people with obesity more prone to reinfection [100]. Moreover, obese mice infected with H1N1 influenza virus showed a diminished number of bone marrow resident B-cells, normally acting as the primary memory cells responsible for antibody production [101, 102]. SARS-CoV-2 infection shares several features of an altered adaptive immune response with obesity. As described above, SARS-CoV-2 infection also causes a significant reduction in circulating lymphocytes and T-cells. Lymphopenia was noted in over 80% of infected patients. Especially decreased numbers of CD4+ and CD8+ T-cells were observed in peripheral blood of COVID-19 patients and were markers for a more severe disease course [103]. The depletion of both CD4+ and CD8+ cells enables a macrophage and neutrophil predominance in the immune response [104]. In contrast, specific T-helper1 (Th1) and T-helper17 (Th17) cells seem to be activated in SARS-CoV-2 and contribute to increased inflammation [105]. Th1 and Th17 cells have pro-inflammatory effects by inducing fibrosis and insulin resistance and are abundantly present in the adipose tissue of people with obesity [106]. It seems likely that the imbalanced immune system connected to obesity exacerbates the immunological derangements of the COVID-19 infection.

## The renin-angiotensin-aldosterone system

**Fig. 3:**
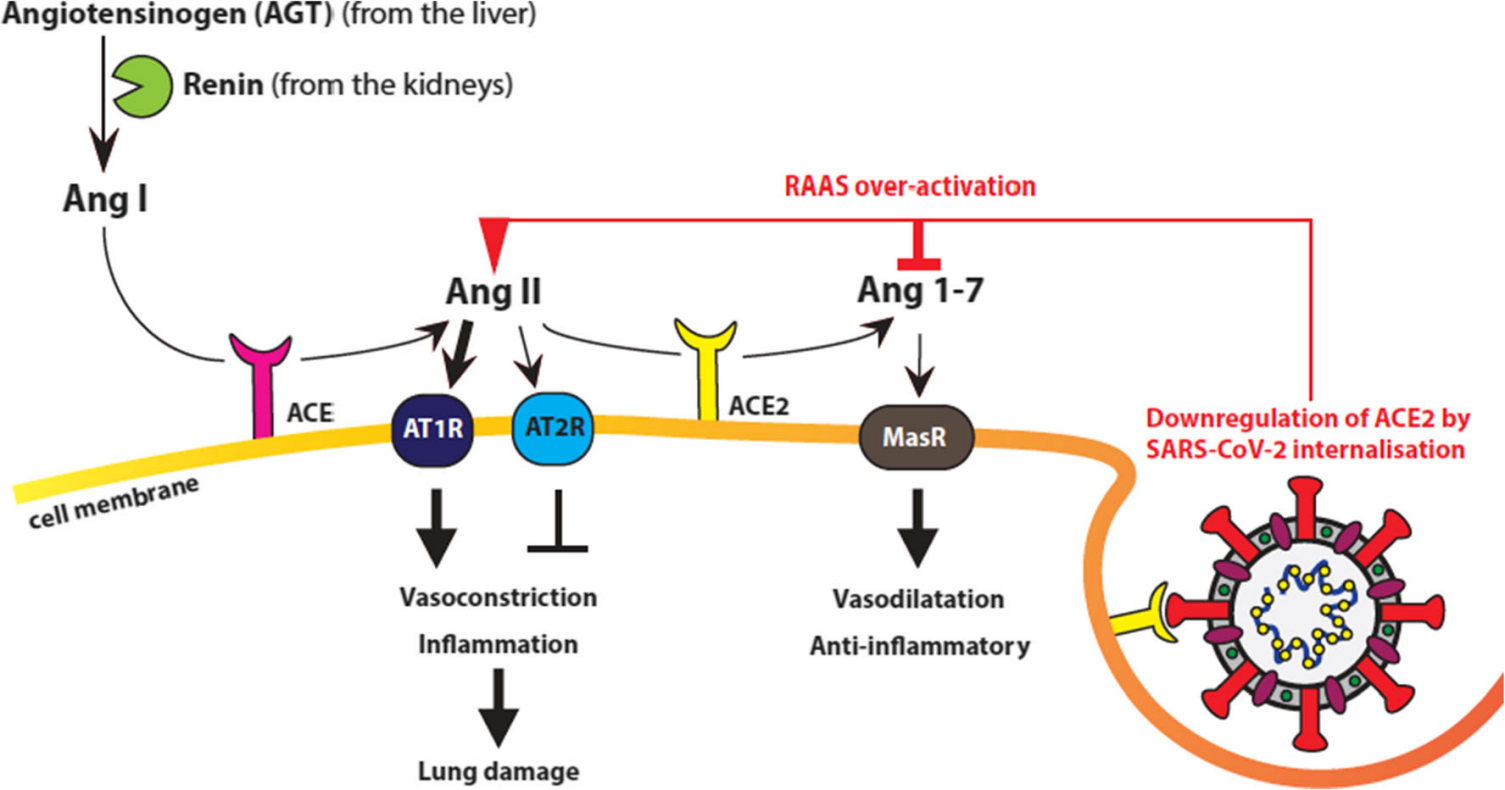
Graphic representation of the RAAS system. Angiotensinogen (AGT) produced by the liver is processed into angiotensin I (Ang I) by renin produced by the kidneys. Ang I is cleaved by angiotensin-converting-enzyme (ACE) to form angiotensin II (Ang II), which binds to Ang II type 1 receptor (AT1R) and to a lesser extend to AT2R. The ACE/AngII/AT1R axis serves as a vasoconstrictor and has potent inflammatory and pro-fibrotic roles triggering lung endothelial dysfunction. ACE2 negatively controls the Ang I/ACE/Ang II/AT1R axis by processing Ang II into Ang 1-7. Ang 1-7 binds to the mitochondrial assembly receptor (MasR) receptor, which results in vasodilatation and has anti-inflammatory and anti-fibrotic effects. Upon infection by SARS-CoV-2 ACE2 is internalized together with the virus resulting in decreased levels of functional ACE2 and in overactivation of the detrimental Ang I/ACE/Ang II/AT1R axis

The SARS-CoV-2 entry receptor ACE2 is a key component of the renin-angiotensin-aldosterone system (RAAS, also called renin-angiotensin system (RAS)), which plays an essential role in maintaining blood pressure, and liquid and electrolyte homeostasis. The RAAS cascade (Fig. 3) begins with angiotensinogen (AGT) produced by the liver and its cleavage/processing into angiotensin I (Ang I) by renin produced by the kidneys. Subsequently, Ang I is cleaved by angiotensin-converting-enzyme (ACE) to form angiotensin II (Ang II), which binds to Ang II type 1 receptor (AT1R) (and to a lesser extend to AT2R). The ACE/AngII/AT1R axis serves as a potent vasoconstrictor, stimulates aldosterone release, and regulates renal sodium reabsorption and potassium excretion. In addition, the ACE/AngII/AT1R axis also has a potent inflammatory and pro-fibrotic role and triggers important adverse reactions such as myocardial hypertrophy and dysfunction, endothelial dysfunction, obesity-associated hypertension, interstitial fibrosis, and oxidative stress [38, 39, 107, 108]. ACE2 is a central factor in the negative control of the Ang I/ACE/Ang II/AT1R axis by processing Ang II into Ang 1-7, and in addition by direct conversion of Ang I into Ang 1-9, which is subsequently processed into Ang 1-7 by ACE or other peptidases. Ang 1-7 binds to the mitochondrial assembly receptor (MasR), which results in vasodilatation, anti-inflammatory, and anti-fibrotic effects. Hence, ACE2 is important for maintaining balance in the RAAS, and higher levels of ACE2 can induce a shift from the inflammatory ACE/AngII/AT1R axis towards the protective ACE2/Ang1-7/Mas axis [107].

ACE2 is highly expressed by alveolar type II pneumocytes, whose function is to produce surfactant, maintain self-renewal, and exert immunoregulatory functions. When a cell is infected by SARS-CoV-2, the S-protein-ACE2 interaction and subsequent membrane fusion lead to internalization of the virus but also of ACE2 and thus result in a decrease in functional ACE2. Less available ACE2 means less Ang II is converted into Ang 1-7. This enhances the ACE/AngII/AT1R axis and its adverse effects and may contribute to pulmonary inflammation and coagulation in COVID-19 disease. Accordingly, the plasma levels of Ang II are markedly elevated in SARS-CoV-2 infected patients and associate linearly to viral load and lung injury [109]. Typically seen in lungs of COVID-19 patients is infiltration of interstitial mononuclear cells such as macrophages and monocytes and chronic inflammatory infiltrates [38, 110] that are believed to contribute to lung damage (see paragraph: COVID-19 immunopathology). This is consistent with the fact that downregulation of ACE2 enhances the adhesion of monocytes and neutrophils to endothelial and mesangial cells through higher Ang II levels [111]. The disturbed balance of the ACE/AngII/AT1R axis in COVID-19 patients might also contribute to the thromboembolic complications often seen in these patients. ACE2 function activates the fibrinolysis inducing plasminogen activator (tPA) [112] while AngII through AT1R stimulates the release of plasminogen activator inhibitor-1 (PAI-1) from endothelial cells that counteracts tPA function and therefore acts prothrombotic [113–115]. Thus, the lower ACE2 and elevated AngII function seen in COVID-19 patients probably shift the tPA/PAI-1 balance towards a prothrombotic state [116]. The connection between the ACE/AngII/AT1R axis and the course of COVID-19 is further supported by the finding that patients with variable degree of ACE2 deficiency associated with, i.e., older age, hypertension, diabetes, cardiovascular disease, seem to develop a more severe disease [39]. Furthermore, the ACE/AngII/AT1R axis is linked with cardiovascular disease, diabetes, and hypertension, which are all associated with higher mortality in COVID-19 [33, 42].

The RAAS pathway is interconnected with the Kallikrein-Kinin-System (KKS), the latter also being involved in inflammatory and thromboembolic disorders [117, 118]. There is some evidence and speculation in the literature suggesting that disturbances due to ACE2 deficiency caused by SARS-CoV-2 infection could be involved in inflammatory and thromboembolic risks of COVID-19 [113]. The KKS consists of the coagulation factor XII (FXII), Prekallikrein (PK), and high molecular weight kininogen (HK). Its activation results in the generation of Bradykinin (BK) that has anti-vasodilating function [119]. BK can be further metabolized to des-Arg^9^-bradikinin (DABK) which is an important pro-inflammatory mediator through activation of a G protein-coupled receptor B1. DABK is also present on airway epithelial cells and it has been suggested that ACE2 cleaves and thereby inactivates DABK which contributes to the anti-inflammatory function of ACE2 [119]. Therefore, the COVID-19 induced ACE2 downregulation probably boosts inflammation via both AngII and DABK.

The RAAS system also plays a major role in the chronic inflammatory state observed in obesity.

White adipose tissue produces the major components of the RAAS-pathway locally and their expression strongly increases upon obesity [120–122]*.* In obese individuals, adipose tissue is actually the most prominent tissue in the body that produces Ang II [123–125]. Ang II is considered to be a major pro-inflammatory adipokine linked to obesity, inflammation, and insulin resistance [120, 123, 126, 127]. It has been shown that Ang II induces the polarization of macrophages towards, pro-inflammatory M1-type macrophages that are involved in RAAS-related disturbances in obese individuals. When they infiltrate in the adipose tissue in response to elevated Ang II and secrete pro-inflammatory cytokines, they contribute to disturbed adipocyte function but also to systemic inflammation [128–130]. Mice AGT overexpression in adipose tissue results in elevated Ang II levels and in increased adipose and systemic inflammation [123, 124]. On the other hand, inactivation of AGT resulted in reduced infiltration of adipose tissues by macrophages, reduced inflammation, and enhanced metabolic activity [131]. In obese, diabetic mice, Ang II blockers lowered systolic blood pressure, and also inhibited adipocyte hypertrophy, suppressed IL-6 expression, and relieved oxidative stress [129].

The increased pro-inflammatory cytokine production due to a dysregulated RAAS homeostasis in WAT of patients with obesity probably contributes to increased severity of COVID-19. In addition, obese and diabetic individuals show reduced ACE2 expression in the vasculature which results in increased vascular permeability and endothelial dysfunction that could lead to complications in patients with a severe COVID-19 development [107, 132].

## Leptin and growth hormone signaling pathways

Leptin is a hormone mainly secreted by the WAT which has various functions in both the endocrine and immune system [86, 133–135]. Leptin secretion can be considered a satiety signal, and its levels rise exponentially with increasing fat mass. Leptin acts by binding to the leptin receptor (LEPR) in the cell membrane of a wide range of cell types throughout the body. Its actions are complex and can be separated into central functions involving the hypothalamus and functions in peripheral tissues. Through its central hormonal function, it affects food intake, physical exercise, energy balance, and adipose tissue mass. Leptin in concordance with other hormones and regulators of energy expenditure indirectly affects insulin sensitivity, and other hormone and cytokine functions. Its crucial role in metabolism has been shown experimentally in lepR-deficient mice models becoming obese due to increased food intake. Similarly, human mutations also result in severe obesity. We refer to several other publications for more in-depth review [136, 137]. Obese individuals develop leptin resistance over time through desensitization of the leptin receptor resulting in increased production and secretion of leptin.

In addition to its role in energy regulation, leptin promotes inflammatory reactions by acting on the leptin receptor present on immune cells of both the innate and adaptive immune system. Leptin increases monocyte and macrophage proliferation and thus increases the level of immune factors and cytokines such as tumour necrosis factor alpha (TNF-α), IL-1, and IL-6 [138, 139]. At the same time IL-1, TNF-α, and lipopolysaccharides (LPS) can increase leptin levels, showing the bidirectional effect that sustains a pro-inflammatory response [139, 140]. These effects contribute to disruption of the immune response [130] [134].

Increased leptin levels of patients with obesity contribute to chronic low-grade inflammation associated with obesity, which is believed to promote several obesity related diseases such as type-2 diabetes, autoimmune diseases, and cardiovascular diseases. In contrast, in malnourished individuals, circulating levels of leptin are low, which suppresses the immune response and makes these people more susceptible to infections [133, 139]. Excessive adipose tissue and high circulating levels of leptin may contribute to severe progression of COVID-19 and to development of respiratory failure and ARDS. A small study demonstrated that COVID-19 patients who needed mechanical ventilation had significantly higher serum/blood leptin levels compared to a control group without the need for mechanical ventilation [86]. These high leptin levels in these SARS-CoV-2 patients correlated with their BMI suggesting that obesity was the underlying cause of the observed high leptin levels and thus of the more serious presentation of the COVID-19 disease. Based on these observations, the hypothesis was mounted that high leptin levels support development of a cytokine storm and (pharmacological) interference with leptin production might be considered a possible treatment of COVID-19 [86].

Growth hormone (GH) is produced in and secreted from the anterior pituitaryy gland. GH stimulates muscle mass, bone density, and bone length during childhood and puberty, and regulates lipid and carbohydrate metabolism during the whole lifespan. GH binds to the growth hormone receptor (GHR) that is expressed mainly in the liver and thereby stimulates the production of the growth factor insulin-like growth factor 1 (IGF1) and -2 (IGF-2). IGF-1 binds and activates the IGF-1 receptor present at the membrane of many different cell types and provokes a growth and proliferation signal [141]. Furthermore, the GH–IGF-1 signaling pathway is crucial for the development and function of the immune system and its disruption can result in immune system impairment as shown in rodent models [142]. It has been demonstrated that GH stimulates the proliferation, differentiation, and survival of antigen-responsive B and T lymphocytes [142] and induces the polarization of macrophages towards the anti-inflammatory M2 type [143]. Strikingly, people with obesity that have a high risk for a severe development of COVID-19 are characterized by strongly reduced GH levels [144]. It is therefore tempting to speculate that the lack of GH in obese patients might contribute to the strong lymphopenia particularly observed in severe cases of the disease [74–76]. Furthermore, reduced GH levels are associated with an increased pro-inflammatory function of macrophages and elevated secretion of inflammatory cytokines like TNFα and IL6 [145], and therefore could promote the inflammatory syndrome that is associated with severe COVID-19. However, the effects of GH deficiency and GH treatment on monocyte/macrophage function and inflammatory cytokine secretion are discussed controversially and GH treatment can also have pro-inflammatory effects [146].

Another complication that is often observed with severe COVID-19 is the occurrence of thrombosis in the venous system and to a lesser extent in the arterial system, which contributes to the increased mortality [147]. GH deficiency can promote thrombosis because it results in an impairment of the fibrinolysis system [148] and therefore could be connected to COVID-19 associated thrombosis. Furthermore, GH levels decrease also during ageing [141] and are generally lower in males compared to females [149], which fits to the strongly increased risk for a severe COVID-19 development in older patients [150] and to the higher risk of male patients [151–153]. Thus, treatment with recombinant human GH could help to activate the adaptive immune system and dampen the severe course of the disease and might be beneficial particularly for obese and older COVID-19 patients that suffer from reduced GH levels [154].

## Obesity and contagiousness

It has been described that virally infected individuals with obesity are more contagious than lean counterparts [90, 155, 156]. For example, the amount of influenza virus in exhaled breath positively correlates with BMI [156]. Several mechanisms contribute to this increased contagiousness. To begin with, viral shedding is prolonged to 104% and thus chances of spreading the virus are increased [155]. Furthermore, the disrupted immune response causes delayed production of interferons which gives the virus the opportunity to replicate more RNA and get more virulent [157, 158]. These findings indicate that a population with a high number of patients with obesity might increase the risk of the appearance of more virulent viruses and will eventually lead to higher mortality overall [90]. COVID-19 is rapidly spreading around parts of the world particularly where obesity prevalence is alarmingly high, for example in the United States of America, where more than 40% of the residents live with obesity (WHO, https:/www.who.int). It is possible that the high prevalence of obesity contributes to the rapid and extensive spread of SARS-CoV-2 in these countries.

## Vaccination and obesity

Host factors such as age, sex, pregnancy, and immune history play an important role in virus vaccine efficacy [159]. Obesity also has an impact on this efficacy; adults with obesity have a two times higher risk, respectively 9.8% compared to 5.1% in leaner counterparts, for developing influenza or influenza-like illnesses after developing efficient antibody response to the given trivalent influenza vaccine [160]. It is thought that lower effectiveness of the influenza vaccine is mediated by poor T-cell function. In obese individuals, the activation of cytotoxic T-cells through mononuclear cells from peripheral blood is decreased and expression of functional markers like granzyme B and IFN-γ is diminished [161]. Obese mice have decreased numbers of B-cells in the bone marrow, which are the most important memory cells responsible for formation of antibodies [162]. In addition, an increased production of non-neutralizing antibodies and a decreased production of influenza-specific antibodies were seen in reaction to an adjuvant influenza vaccine [163]. It was suggested that chronic inflammation in obesity might play a role in the reduced efficacy of inactivated monochronic influenza vaccine [164]. Increasing the dose or using an adjuvant was not efficient when obese mice had been infected with the influenza virus [163]. Another study aimed to compare the serologic response to a monovalent H1N1 influenza vaccine in children and adults of various BMI. After a single dose of H1N1 vaccine, higher geometric mean titers (GMT) of hemagglutination inhibition antibody (HAI) were seen in adults with obesity compared to leaner counterparts, even after correcting for age, race, and pre-vaccination [165]. No difference was seen in serologic response after two doses [161, 165, 166]. After 12 months post-vaccination, obese individuals had significantly lower activation of CD4+ and CD8+ T-cells when challenged with H1N1 ex vivo [161, 167]. Hence, these findings suggest that developing an efficient immune response is not enough to ensure long-term protection in people with obesity.

## Discussion and conclusion

Here we have reviewed the literature on COVID-19 and its relation to obesity as a major risk factor for severe disease development. A meta-analysis assessing COVID-19 fatality rates clearly reveals age as the foremost risk factor. The study demonstrates that differences in the population age structure and age-specific incidence of severe COVID-19 explain nearly 90% of the geographical variation in population infection-related fatality rates (IFR; the proportion of deaths among confirmed cases of the disease). The analysis shows an exponential relationship between age and IFR with very low values for children and younger adults, progressively increasing to 0.4% at age 55, 1.3% at age 65, 4.2% at age 75, and 14% at age 85 [168]. COVID-19 appears to affect men more severely than women with higher mortality rates [151–153, 169]. A combination of different factors, both biological and environmental, likely contributes to the gender difference. For example, plasma levels of C reactive protein (CRP), ferritin, and other inflammatory markers are significantly higher in male patients. This points to a heightened inflammatory response in men with COVID-19. Different mechanisms contributing to this heightened inflammatory response have been proposed like lower ACE2 levels in males compared to females which have been associated with a more severe course of infection or the testosterone-induced expression of the transmembrane protease, serine 2 (TMPRSS2) that is involved in SARS-CoV-2 spike protein priming and cell fusion. Furthermore, data from mouse models suggest a protective function of estrogens in COVID-19 infection through suppression of monocyte–macrophage recruitment [169]. Another meta-analysis revealed several comorbidities for developing severe COVID-19, including obesity, cardiovascular disease, diabetes, respiratory disease (including severe asthma), a history of hematological malignancy or recent other cancer, kidney, liver, and neurological diseases, and autoimmune conditions [170]. Metabolic disturbances superimposed on age-related immunological decline would explain most of the COVID-19 cases with severe course of the disease. Since responding (naïve) T-cells are reduced by age or through immunological derangements caused by obesity-related systemic inflammation, the compromised immune system fails to adequately mount an effective T-cell response upon infection with SARS-CoV-2. In addition, the prevailing innate immune response in the lungs causes a misbalance in inflammatory cytokine secretion that might be reinforced by the chronic inflammation associated with ageing and/or obesity resulting in a cytokine storm syndrome, which causes tissue damage. Particularly, the high circulating levels of the pro-inflammatory adipokine leptin in patients with obesity together with the reduced expression of the anti-inflammatory-acting ACE2 receptors in the lung epithelium of infected patients counteract the clearance of the innate immune response with fatal consequences for the patients. With the use of known anti-inflammatory-acting drugs like glucocorticoids and/or IL-6 inhibitors, it might be able to suppress the overacting innate immune system, which was shown to be beneficial at least in a subset of hospitalized COVID-19 patients. At the end, vaccination will be the most effective measure in the battle against the SARS-CoV-2 virus and its associated complications. However, also here the high-risk patients like the elderly and those with obesity might have the disadvantage of a less efficient immune response and/or a shorter lasting immune memory that limit the vaccination effectivity.
